# New Operation for Internal Hæmorrhoids

**Published:** 1886-05

**Authors:** J. M. Lewis

**Affiliations:** Mexia, Texas


### NEW OPERATION FOR INTERNAL HEMORRHOIDS.

*By J. M. Lewis, M. D. Mexia, Texas.*

(For Daniel's Texas Medical Journal.)

I OPERATED on a man during the month of December, 1885, for internal hemorrhoids. I had prepared my patient by using a purgative the night before, and using an injection of warm water and soap the morning of the operation, as is usual in such cases.

It was my intention to inject the tumors with carbolic acid and olive oil, (equal parts) as I have often done. The acid and oil were warmed, and my hypodermic syringe in good working order; but before I could use it, the mixture became cool, (the weather was very cold) and the syringe would not work well, and in attempting to use it, I carelessly spilled the acid and oil over the tumors. I then abandoned the syringe and used a ligature, and to my surprise, it *did not cause any pain.* The oil and acid had produced local anaesthesia. The operation was a success.

I have operated many times for internal hemorrhoids, and I prefer the ligature to the injection, but the ligature causes pain, while the injection (if properly used) does not.

The ligature is without doubt the safest, surest and best operation, but the pain is some times severe, especially if the ligature is not tied very tight. Now if we can produce *anæsthesia* with the acid and oil then this objection is avoided.

When called upon to operate again, I shall paint the tumors with a solution of carbolic acid and oil before applying the ligature. I hope others may try this method, and while "one swallow don't make a summer," I feel confident it will succeed. We know that carbolic acid is an anæsthetic, and the oil prevents it from acting as a caustic.

In this operation we want a quick anaesthetic, and I believe in carbolic acid, we have it. And besides, I think the tumors will slough quicker.

I am not aware of any one having used carbolic acid in this manner, and as stated in this case, it was an accident.

